# Effects of Bacterial Culture and Calcium Source Addition on Lead and Copper Remediation Using Bioinspired Calcium Carbonate Precipitation

**DOI:** 10.3389/fbioe.2022.889717

**Published:** 2022-05-02

**Authors:** Zhong-Fei Xue, Wen-Chieh Cheng, Lin Wang, Shaojie Wen

**Affiliations:** ^1^ School of Civil Engineering, Xi’an University of Architecture and Technology, Xi’an, China; ^2^ Shaanxi Key Laboratory of Geotechnical and Underground Space Engineering (XAUAT), Xi’an, China

**Keywords:** bioinspired calcium carbonate precipitation, *Sporosarcina pasteurii*, pH, urea hydrolysis, remediation efficiency

## Abstract

Lead and copper ions from wastewater induced by metallurgical processes are accumulated in soils, threatening plant and human health. The bioinspired calcium carbonate precipitation is proven effective in improving the cementation between soil particles. However, studies on capsulizing heavy metal ions using the bioinspired calcium carbonate precipitation are remarkably limited. The present study conducted a series of test tube experiments to investigate the effects of bacterial culture and calcium source addition on the remediation efficiency against lead and copper ions. The calcium carbonate precipitation was reproduced using the Visual MINTEQ software package to reveal the mechanism affecting the remediation efficiency. The degradation in the remediation efficiency against lead ions relies mainly upon the degree of urea hydrolysis. However, higher degrees of urea hydrolysis cause remediation efficiency against copper ions to reduce to zero. Such high degree of urea hydrolysis turns pH surrounding conditions into highly alkaline environments. Therefore, pursuing higher degrees of urea hydrolysis might not be the most crucial factor while remedying copper ions. The findings shed light on the importance of modifying pH surrounding conditions in capsulizing copper ions using the bioinspired calcium carbonate precipitation.

## Introduction

Metallurgical processes, metal smelting activities, and inappropriate wastewater disposal discharge heavy metals into soils and groundwater, hence resulting in serious ecological environmental pollution (Nakata et al., 2017; Vital et al., 2018; Bai et al., 2021a; Bai et al., 2021b; Bai et al., 2021d; Xue et al., 2021a; Xue et al., 2021b; Wang et al., 2021; Chen et al., 2022). Lead (Pb) and copper (Cu), that are featured with characters of toxicity, non-biodegradability, and bioaccumulation, are considered two common and dangerous heavy metal contaminants present in surrounding environments, which cause serious threats to plant and human health (Zhao et al., 2016; Jiang et al., 2019; Liao et al., 2020). The mobility or solubility of heavy metals can transform them from the solid phase to the solution phase, thereby increasing their bioavailability (Bolan et al., 2014; Bai et al., 2020, Bai et al., 2021c; Yang et al., 2021; Xue et al., 2022). Therefore, immobilizing heavy metals is considered to be of great necessity in order to reduce their bioavailability (Li et al., 2013; Khadim et al., 2019; Cheng et al., 2021; Hu et al., 2021; Hu et al., 2022; Wang et al., 2022; Wang et al., 2022; Wang et al., 2022). To this end, chemical precipitation, electroremediation, and ion-exchange and adsorption have been widely adopted as countermeasures. Notwithstanding that, these methods are neither effective nor economical and may cause secondary environmental contamination (Bhattacharya et al., 2018; Zhao et al., 2020). Bioprecipitation of calcium carbonate has recently gained much attention from scientific researchers and industrial participants, which is proven economic and effective for immobilizing heavy metals and substantially reduces the potential of secondary environmental contamination (Rahman et al., 2020; Rajasekar et al., 2021).

The fundamental principle of applying the bioinspired calcium carbonate precipitation technology to the immobilization of heavy metals is to catalyze urea hydrolysis using the ureolytic bacteria toward discharging hydroxide ions and ammonium ions and precipitating calcium carbonate with heavy metals (Schwantes-Cezario et al., 2017; Qiao et al., 2019; Schwantes-Cezario et al., 2020; Yang et al., 2020; Hu et al., 2021a; Hu et al., 2021b; Yuyang Zhang et al., 2021; Yuan et al., 2021). The whole process is also referred to as “biomineralization”, and heavy metal migration potential can be substantially reduced when capsulized using the calcium carbonate precipitation, minimizing their threats to surrounding environments (Zhu and Maria, 2016; Arias et al., 2019; Teng et al., 2019; Song et al., 2021) (see Eqs 1–7). Jiang et al. (2019) reported that calcium source and bacterial concentration can affect the remediation efficiency against lead and three mechanisms, namely, abiotic precipitation, biotic precipitation, and biosorption, which take part in the biomineralization process. Qiao et al., 2021 declared that the bioinspired calcium carbonate precipitation technology is effective in removing heavy metals such as Cu, Zn, Ni, and Cd.

$$\mathrm{CO}{\left(NH_{2}\right)}_{2}+H_{2}O\to 2NH_{3}+CO_{2}.$$ (1)

$$2NH_{3}+2H_{2}O↔2NH_{4}^{+}+2OH^{-}.$$ (2)

$$CO_{2}+2\mathrm{OH}↔\mathrm{HC}O_{3}^{-}+OH^{-}↔CO_{3}^{\mathrm{2-}}+H_{2}O.$$ (3)

$$Ca^{2+}+\mathrm{Cell}\to \mathrm{Cell}-Ca^{2+}.$$ (4)

$$M^{2+}+\mathrm{Cell}\to \mathrm{Cell}-M^{2+}.$$ (5)

$$\mathrm{Cell}-Ca^{2+}+CO_{3}^{2-}\to \mathrm{Cell}-\mathrm{CaC}O_{3}\left(s\right).$$ (6)

$$\text{Cell}-{\text{M}}^{2+}+\text{C}{\text{O}}_{3}^{2-}\to \text{Cell}-\text{MC}{\text{O}}_{3}\left(\text{s}\right).$$ (7)

A ratio of the removed heavy metal concentration to the initial concentration has been defined as remediation efficiency, and it may vary with the degree of urea hydrolysis, pH surrounding conditions, and concentration of heavy metal ions (Achal et al., 2012; Xue et al., 2022). As reported by Duarte-Nass et al. (2020), the remediation efficiency against copper degrades with the decrease in the degree of urea hydrolysis, and the degradation becomes more pronounced when subjected to higher heavy metal concentrations. Mugwar and Harbottle (2016) found that elevation of pH surrounding conditions and calcium carbonate precipitation is shown to be strongly linked to the removal of zinc and cadmium. Although a significant body of research has investigated the degree of urea hydrolysis and its link to remediation efficiency, how they vary with the effect of bacterial culture remains unclear (Torres-Aravena et al., 2018; Seplveda et al., 2021; Shi et al., 2022). Furthermore, given that calcium source addition takes part in precipitating calcium carbonate, its implications on the ureolytic bacteria and urease activity are, however, rarely studied. The main objectives of the present study are as follows: 1) to investigate the effect of bacterial culture and calcium source addition on the remediation efficiency against Pb^2+^ and Cu^2+^ and 2) to reveal the mechanism affecting remediation efficiency.

## Materials and Methods

### Bacteria and Cultivation

*Sporosarcina pasteurii* in a freeze-dried form from China General Microbiological Culture Collection Center (CGMCC) was used as ureolytic bacteria for catalyzing urea hydrolysis. The strain was transferred to a liquid medium which consists of yeast extract (Oxoid Ltd., United Kingdom) of 20 g/L, urea (Damao Chemical Reagent Factory, China) of 20 g/L, NH_4_Cl (Chengdu Chron Chemicals Co. Ltd., China) of 10 g/L, MnSO_4_·H_2_O (Shanghai Aladdin Biochemical Technology Co. Ltd., China) of 10 mg/L, and NiCl·6H_2_O (Tianli Chemical Reagent Co. Ltd., China) of 24 mg/L and was cultured at 180 rpm and 30°C for 24 h. pH for the liquid medium was measured being 8.8. The activated ureolytic bacteria were mixed with glycerol using a ratio of 7:3 and stored at −20°C (Achal et al., 2012). They were used as the inoculum in subsequent test tube experiments.

### Test Tube Experiments

The purpose for implementing the test tube experiments is to reproduce the biomineralization process using the ureolytic bacteria for Pb and Cu immobilization. The testing scheme for the test tube experiments under the effect of bacterial culture (i.e., yeast extract) and calcium source (i.e., CaCl_2_) addition is summarized in Table 1. Bacterial solution from the frozen stock was first inoculated (0.1% (v/v)) into the liquid medium containing Cu(NO_3_)_2_ or Pb(NO_3_)_2_ at concentrations varying in a 0–50 mM range, 0.5 M urea, 2 g/L yeast extract, and 0.25 M CaCl_2_. There were three replicates for each testing set. Urease activity (UA) and pH measurements were conducted at 0, 4, 12, 24, and 48 h, while NH_4_^+^ and Pb^2+^ or Cu^2+^ concentration were measured at 0, 24, and 48 h, respectively. pH was measured using a bench pH meter (Hanna Instruments Inc. HI 2003), while UA was measured on a basis of the ureolysis rate, as recommended by Whiffin et al. (2007); 2 ml reaction solution is mixed with 18 ml 1.11 M urea, and EC is measured at 0 and 5 min after mixing. UA can be evaluated using the equation below:

$$UA=\frac{EC_{5}-EC_{0}}{5}\times 10\times 1.11\left(\mathrm{mM}\;\mathrm{Urea}\;\min^{-1}\right),$$ (8)

where *EC*_0_ and *EC*_5_ are electrical conductivity at 0 and 5 min, respectively. Furthermore, NH_4_^+^ concentration in solution was determined using the modified Nessler method (Whiffin et al., 2007). Moreover, Pb^2+^ or Cu^2+^ concentration was measured using an atomic spectrophotometer (Beijing Purkinje General Instrument TAS-990). The remediation efficiency can be evaluated as follows:

$$Remediation\;efficiency=\frac{C_{1}-C_{0}}{C_{0}}\times 100\%,$$ (9)

where *C*_0_ and *C*_1_ are Pb^2+^ or Cu^2+^ concentration before and after remediation, respectively. NH_4_^+^ concentration of the reaction solution is measured at 0.1, 24, and 48 h, and the method for measuring NH_4_^+^ concentration corresponds to the modified Nessler method (Whiffin et al., 2007). Prior to the measurement, a calibration line was set up, and the absorbance was measured using a spectrophotometer 10 min after adding Nessler’s reagent and potassium sodium tartrate. It was substituted into the calibration line, yielding NH_4_^+^ concentration. The more the NH_4_^+^ produced, the higher the degree of urea hydrolysis. For this reason, the NH_4_^+^ concentration was adopted here toward assessing the degree of urea hydrolysis.

**TABLE 1:** Scheme applied to the test tube experiments.

	Bacterial inoculation proportion	Concentration of yeast extract (g/L)	Concentration of CaCl_2_ (mM)	Concentration of Cu(NO_3_)_2_/Pb(NO_3_)_2_ (mM)
Exp-01	1:9	0	0	0, 5, 10, 20, 30, 40, and 50
Exp-02	1:9	20	0	
Exp-03	1:9	20	0.25	

### Biomineralization Simulation

Given that the speciation and sequence of carbonate precipitation cannot be revealed by the experimental results, they were reproduced using the Visual MINTEQ software package. In the present work, urea hydrolysis was modeled using the ratio of NH_4_^+^ to CO_3_^2−^ being 2:1, although the bacterial culture and inoculation were omitted in the numerical simulation (Gat et al., 2017). NH_4_^+^ and CO_3_^2−^ concentrations that are extracted upon the completion of urea hydrolysis aim to backanalyze the speciation and sequence of carbonate precipitation. The details in terms of the numerical simulation are summarized in Table 2. The simulated results are beneficial to analyze how the speciation and sequence of carbonate precipitation vary under the sole effect of yeast extract and the effect of yeast extract and calcium source addition.

**TABLE 2:** Summary of the numerical simulations.

CaCl_2_ (mM)	Concentration of each ions (mM)					
	Cu^2+^/Pb^2+^	NO_3_^−^	NH_4_^+^	CO_3_^2-^	Cl^−^	Ca^2+^
0	5, 10, 20, 30, 40, and 50	10, 20, 40, 60, 80, and 100	33.3–1,033.3	0–500	33.3	0
0.25					533.3	250

## Results and Discussion

### Effect of Bacterial Culture and Calcium Source Addition

**FIGURE 1:**
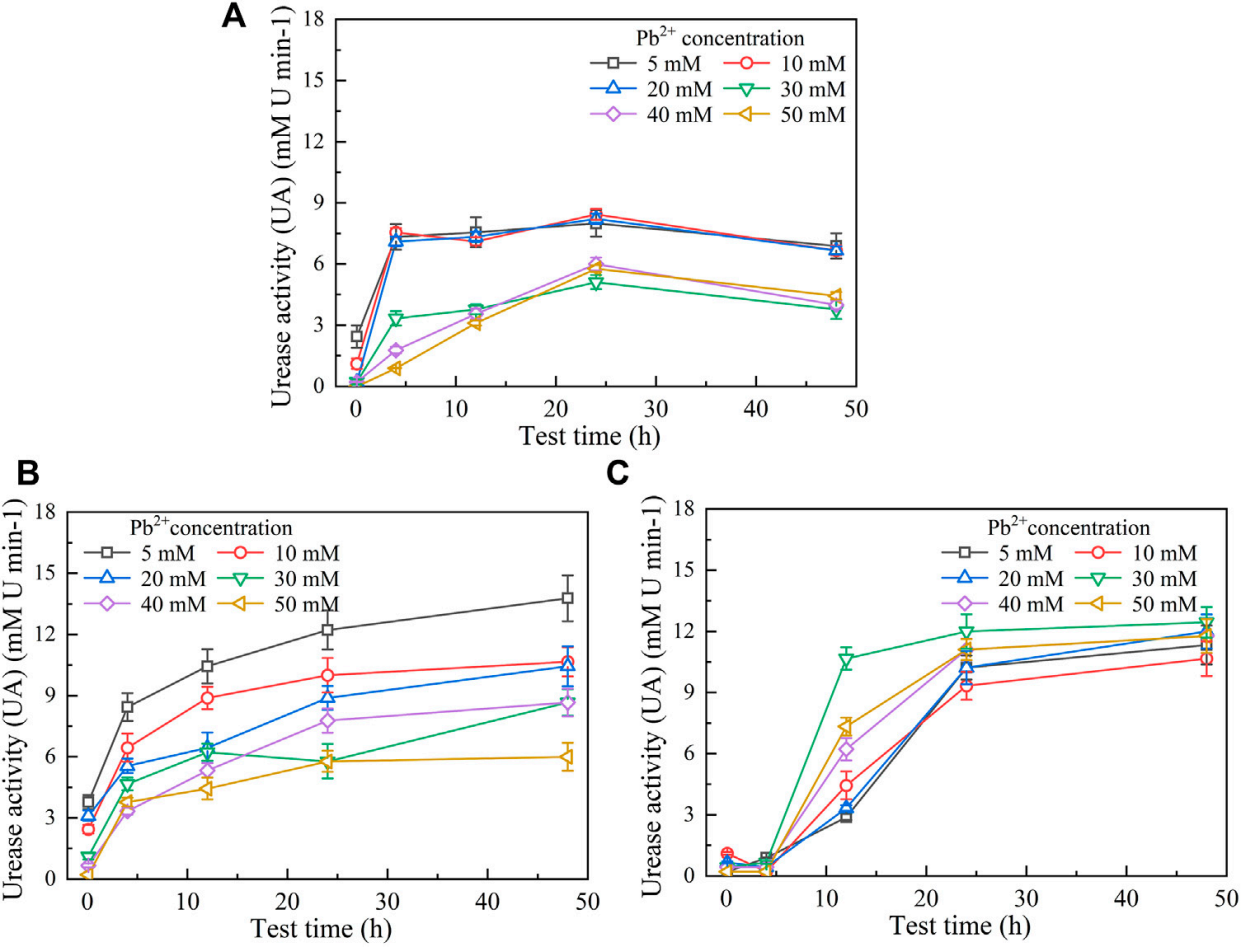
Temporal relationships of urease activity (UA) against Pb(NO_3_)_2_ concentrations: **(A)** without yeast extract and CaCl_2_ addition, **(B)** under the effect of yeast extract, and **(C)** under the effect of yeast extract and CaCl_2_ addition.

UA and pH are deemed as the key indicators that reflect the degree of urea hydrolysis and remediation efficiency while introducing the microbial-induced carbonate precipitation (MICP) technology for heavy metal immobilization (Stocks-Fischer et al., 1999; Zhu and Maria, 2016; Omoregie et al., 2017). This section aims to analyze the effect of bacterial culture and calcium source addition on the degree of urea hydrolysis during the MICP process. As shown in Figures 1A–C, UA generally decreases with the increase of Pb(NO_3_)_2_ concentration. The temporal relationships, when not subjected to yeast extract and CaCl_2_ addition, can be characterized as UA ascending initially and descending after reaching the peak (Figure 1A). Furthermore, the temporal relationships of UA are divided into two curve groups; the temporal relationships of UA under the Pb(NO_3_)_2_ concentration ≤20 mM lie above those under the Pb(NO_3_)_2_ concentration >20 Mm. When subjected to the effect of yeast extract, the temporal relationships of UA are found to occur in an ascending manner throughout (Figure 1B). The temporal relationships under the effect of yeast extract and CaCl_2_ addition can be characterized as UA descending initially and ascending after reaching the minimum value (Figure 1C).

The secretion of urease by the ureolytic bacteria is determined by whether the availability of sufficient nutrients can be assured for bacterial growth and reproduction. Higher concentrations of Pb^2+^ prevent the ureolytic bacteria from secreting and producing enough extracellular polysaccharides (EPS), aggravating the effect of Pb^2+^ and deactivating the ureolytic bacteria (Wu et al., 2019). On the other hand, higher Pb^2+^ concentrations can also cause urease denaturation, leading to its deactivation. These results, in turn, impede the development of UA. The consumption of nutrients over time is the main contributor to the formation of the descending tendency of bacterial activity. For this reason, UA descends slowly after 24 h (Figure 1A). In contrast, the ureolytic bacteria under the effect of yeast extract continuously secrete urease, and therefore, UA increases all along (Figure 1B). Considering CaCl_2_ is a calcium source addition and the yeast extract aids the ureolytic bacteria to secrete urease, the negatively charged ureolytic bacteria adsorb Pb^2+^ and Ca^2+^ and precipitate with CO_3_^2-^ when using the ureolytic bacteria as nucleation sites (Rui Zhang et al., 2021; Yin et al., 2021). To this end, the ureolytic bacteria settle together with the carbonate precipitation toward the bottom of the test tube, reducing the bacterial concentration in the supernatant and subsequently reducing UA (Figure 1C). Thus, UA shows a decreasing tendency from 0.1 to 4 h . Ca^2+^ form competitive adsorption with Pb^2+^ and cause the ureolytic bacteria to preferentially bind themselves together with Ca^2+^, which also indicates an enhancement of the resistance against Pb^2+^. Given the effect of CaCl_2_ addition, the ureolytic bacteria that commence showing their resistance against the effect of Pb^2+^ continue to grow and secrete urease, and therefore, UA increases after reaching the minimum from 4 to 48 h. This shows a difference in the curve slopes from those we have seen in Figure 1B.

**FIGURE 2:**
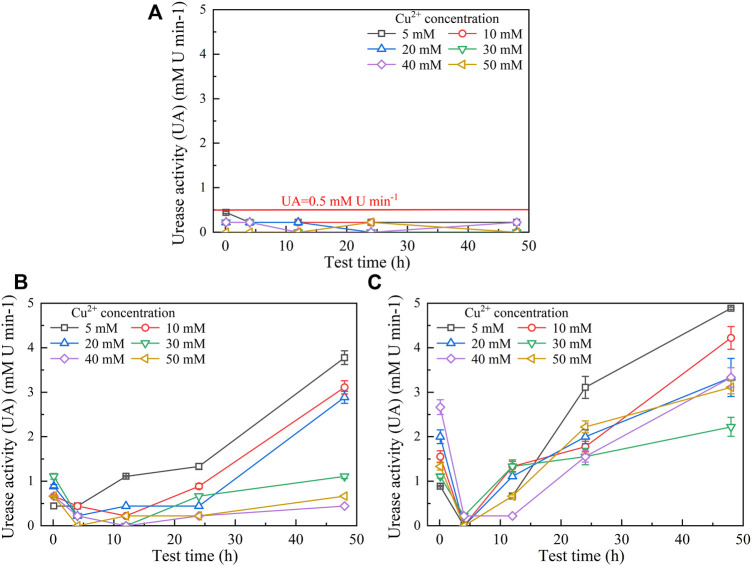
Temporal relationships of pH against Pb(NO_3_)_2_ concentrations: **(A)** without yeast extract and CaCl_2_, **(B)** under the effect of yeast extract, and **(C)** under the effect of yeast extract and CaCl_2_ addition.

The temporal relationships of pH in the mixture against Pb(NO_3_)_2_ concentrations are shown in Figure 2. They can be characterized with the pH increasing rapidly at the very beginning of the MICP process and then remaining constant or decreasing slightly after reaching the peak. Furthermore, similar to the temporal relationships of UA, pH decreases with the increase of Pb(NO_3_)_2_ concentration. Moreover, when subjected to no yeast extract and CaCl_2_ addition, pH reaches its maximum value being approximately 9.5 at 12 h after the commencement of the MICP process (Figure 2A). pH, when subjected to the effect of yeast extract, reaches its maximum of about 8.5 after 12 h, which is close to that under the effect of yeast extract and CaCl_2_ addition (Figures 2B,C). These results can help infer that under the sole effect of yeast extract, a small number of ureolytic bacteria remain active when subjected to lower Pb^2+^ concentrations and the majority of the ureolytic bacteria lose their activity when subjected to higher Pb^2+^ concentrations. The smaller the number of ureolytic bacteria remaining active, the lower the degree of ureo hydrolysis. Therefore, UA distributes in a scattered manner at the end of the process, and due to the lack of NH_4_^+^ resulting from the reduced degree of urea hydrolysis, pH reduces to 8.5. pH reducing to 8.5 could also be ascribed to bacteria metabolism. Bacteria metabolism consumes carbon from the yeast extract, yielding organic and carbonic acids and lowering pH (Jimenez-Lopez et al., 2008). In contrast, UA is beyond 10 at the end of the process under the effect of yeast extract and CaCl_2_ addition. This is because the majority of the ureolytic bacteria commence showing their resistance against the effect of Pb^2+^ (Peng et al., 2021). The higher degree of urea hydrolysis facilitates the formation of carbonate precipitation, which also indicates consumption of CO_3_^2−^ and further decrease in pH.

**FIGURE 3:**
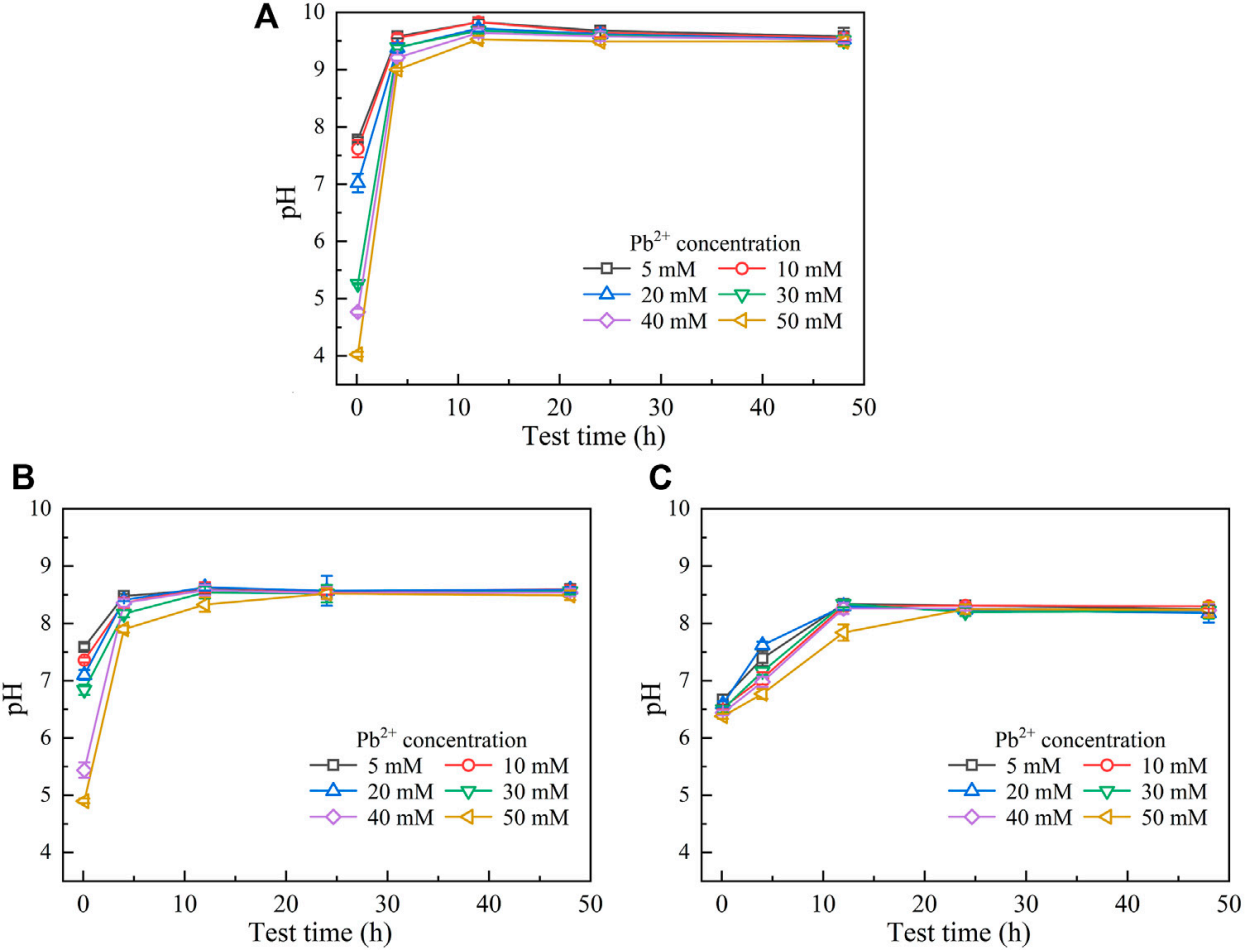
Temporal relationships of urease activity (UA) against Cu(NO_3_)_2_ concentrations: **(A)** without yeast extract and CaCl_2_, **(B)** under the effect of yeast extract, and **(C)** under the effect of yeast extract and CaCl_2_ addition.

**FIGURE 4:**
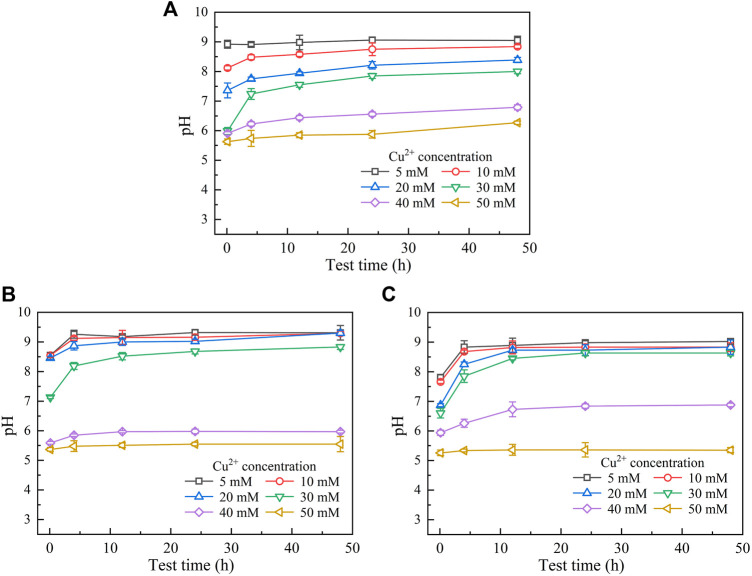
Temporal relationships of pH against Cu(NO_3_)_2_ concentrations: **(A)** without yeast extract and CaCl_2_, **(B)** under the effect of yeast extract, and **(C)** under the effect of yeast extract and CaCl_2_ addition.

Figures 3, 4 show the temporal relationships of UA and pH against Cu(NO_3_)_2_ concentrations, respectively. UA = 5 mM U min^−1^ (maximum) under the effect of Cu^2+^ increases to UA = 13 mM U min^−1^ (maximum) under the effect of Pb^2+^ (Figures 1, 3), indicating the effect of Cu^2+^ on the bacterial activity outweighing the effect of Pb^2+^. For this reason, UA under the effect of Cu^2+^ could be as low as 0.5 mM U min^−1^ (Figure 3A). The effect of Cu^2+^ depresses the ureolytic bacteria and then UA, as indicated by the descending tendency from 0 to 4 h (Figure 3B). The effect of yeast extract aids the ureolytic bacteria to secrete urease and promotes notably the development of UA. This especially holds true when subjected to lower Cu^2+^ concentrations and is considered the main cause, leading to a discrepancy in the curve slope after 24 h. As indicated by the measurements of UA, when the ureolytic bacteria provide nucleation sites for precipitating CaCO_3_, the concentration of the ureolytic bacteria in the supernanant is reduced and the development of UA is depressed, forming the descending tendency from 0 to 4 h (Figure 3C). The addition of CaCl_2_ not only promotes the formation of CaCO_3_ but also develops resistance of the ureolytic bacteria against the effect of Cu^2+^. Such resistance is especially significant when subjected to lower Cu^2+^ concentrations, which also indicates the main cause leading to the difference of the curve slope after 4 h. On the other hand, the lower the Cu^2+^ concentration, the higher the degree of urea hydrolysis, and the higher the concentration of NH_4_^+^ and CO_3_^2-^. The higher concentrations of NH_4_^+^, induced by Cu^2+^ = 5 mM, turn pH surrounding conditions into a strong alkaline environment (i.e., pH = 9), while due to Cu^2+^ = 50 mM, the lower concentrations of NH_4_^+^ turn pH surrounding conditions to a weak acid environment (i.e., pH below 6) (Figure 4A). The effect of yeast extract differentiates pH under higher Cu^2+^ concentrations from those under lower Cu^2+^ concentrations (Figure 4B). There appear two groups; one is pH above 7 and the other is pH below 6. The effect of yeast extract and CaCl_2_ addition, in turn, eases such difference in pH (Figure 4C).

### Evaluation of Remediation Efficiency

**FIGURE 5:**
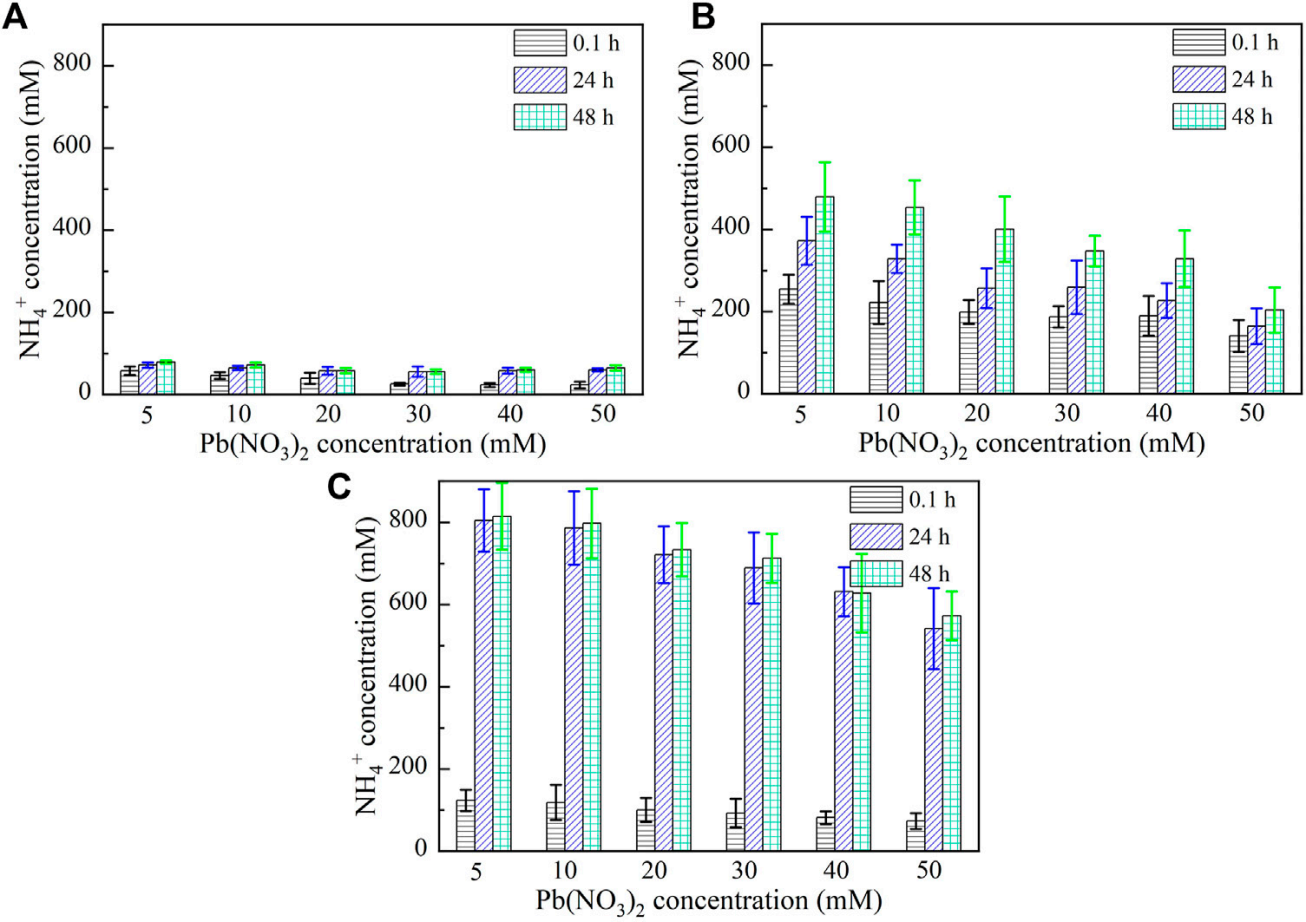
Relationships of NH_4_^+^ concentration versus Pb(NO_3_)_2_ concentration: **(A)** without yeast extract and CaCl_2_, **(B)** under the effect of yeast extract, and **(C)** under the effect of yeast extract and CaCl_2_ addition.

**FIGURE 6:**
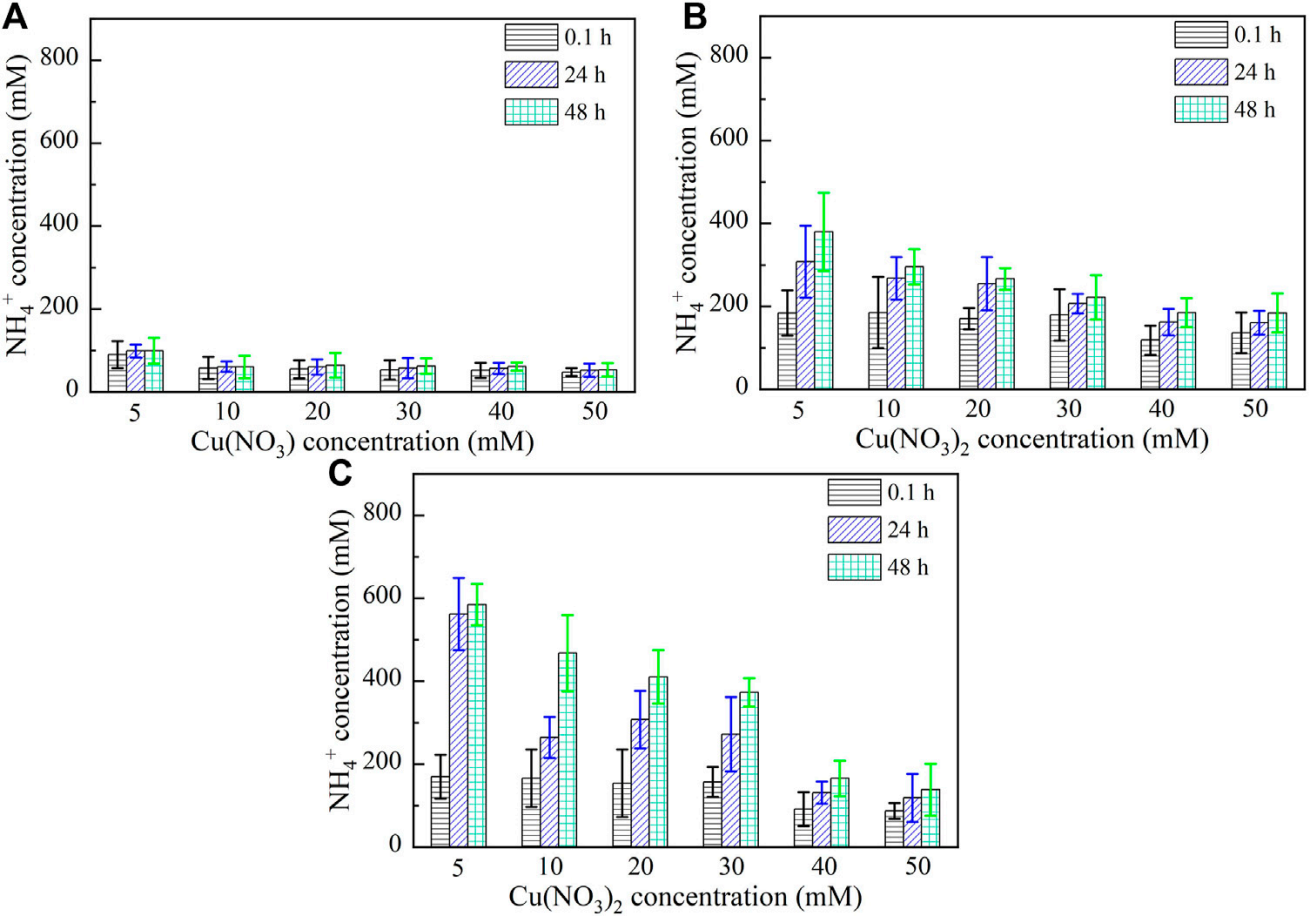
Relationships of NH_4_^+^ concentration versus Cu(NO_3_)_2_ concentration: **(A)** without yeast extract and CaCl_2_, **(B)** under the effect of yeast extract, and **(C)** under the effect of yeast extract and CaCl_2_ addition.

Figures 5, 6 show the variations of NH_4_^+^ concentration against Pb^2+^ and Cu^2+^ concentrations, respectively, after 0.1, 24, and 48 h. It is evident that when subjected to no yeast extract and calcium source addition, the higher the concentration of Pb^2+^ or Cu^2+^, the lower the degree of urea hydrolysis, and the lower the concentration of NH_4_^+^ (Figure 5A and Figure 6A). Furthermore, the concentration of NH_4_^+^ remains nearly unchanged throughout the process, indicating that the majority of the ureolytic bacteria lose their activity when subjected to the effect of Pb^2+^ or Cu^2+^, and this leads to the inability of discharging NH_4_^+^ and CO_3_^2−^ while catalyzing urea hydrolysis. Under the effect of yeast extract, the concentration of NH_4_^+^ shows a significant change with time, indicating a continuous discharge of NH_4_^+^ and CO_3_^2−^ (Figures 5B, 6B). This result also indicates that the yeast extract can ease the effect of Pb^2+^ or Cu^2+^ and prevent the ureolytic bacteria from losing their activity. It is worth noting that the more significant change in NH_4_^+^ concentration under the effect of Pb^2+^ than under the effect of Cu^2+^ provides testimony of the effect of Cu^2+^ outweighing the effect of Pb^2+^. When the ureolytic bacteria, under the effect of yeast extract and CaCl_2_ addition, start showing their resistance against the effect of Pb^2+^ or Cu^2+^, the concentration of NH_4_^+^ after 0.1 h differs notably from that after 24 and 48 h (Figures 5C, 6C); the concentration of NH_4_^+^, under Pb^2+^ = 5 mM, increases quickly from approximately 110 mM after 0.1 h to about 800 mM after 48 h, while under Cu^2+^ = 5 mM, the concentration of NH_4_^+^ increases from about 190 mM after 0.1 h to approximately 600 mM after 48 h. Although the addition of CaCl_2_ promotes the precipitation of CaCO_3_ and depresses the ureolytic bacteria at the very beginning of the process (i.e., after 0.1 h), it enhances the resistance of the ureolytic bacteria against the effect of Pb^2+^ or Cu^2+^ after 24 and 48 h. This is deemed as the main cause leading to the exaggerated difference between the concentration of NH_4_^+^ after 0.1 h and the concentration of NH_4_^+^ after 24 and 48 h.

**FIGURE 7:**
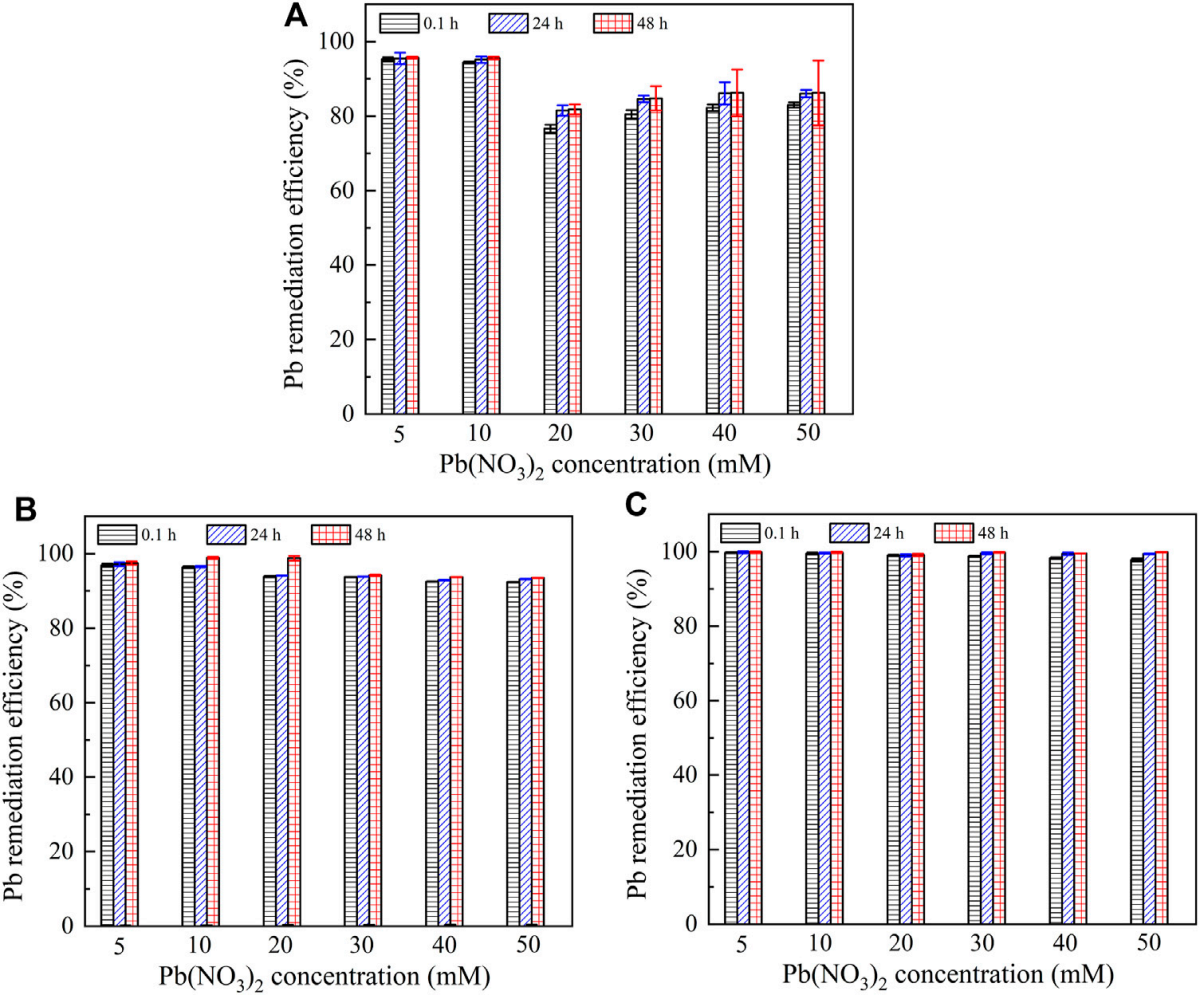
Relationship of remaining Pb^2+^ concentration and Pb remediation efficiency versus Pb(NO_3_)_2_ concentration: **(A)** without yeast extract and CaCl_2_, **(B)** with yeast extract, and **(C)** with yeast extract and CaCl_2_.

**FIGURE 8:**
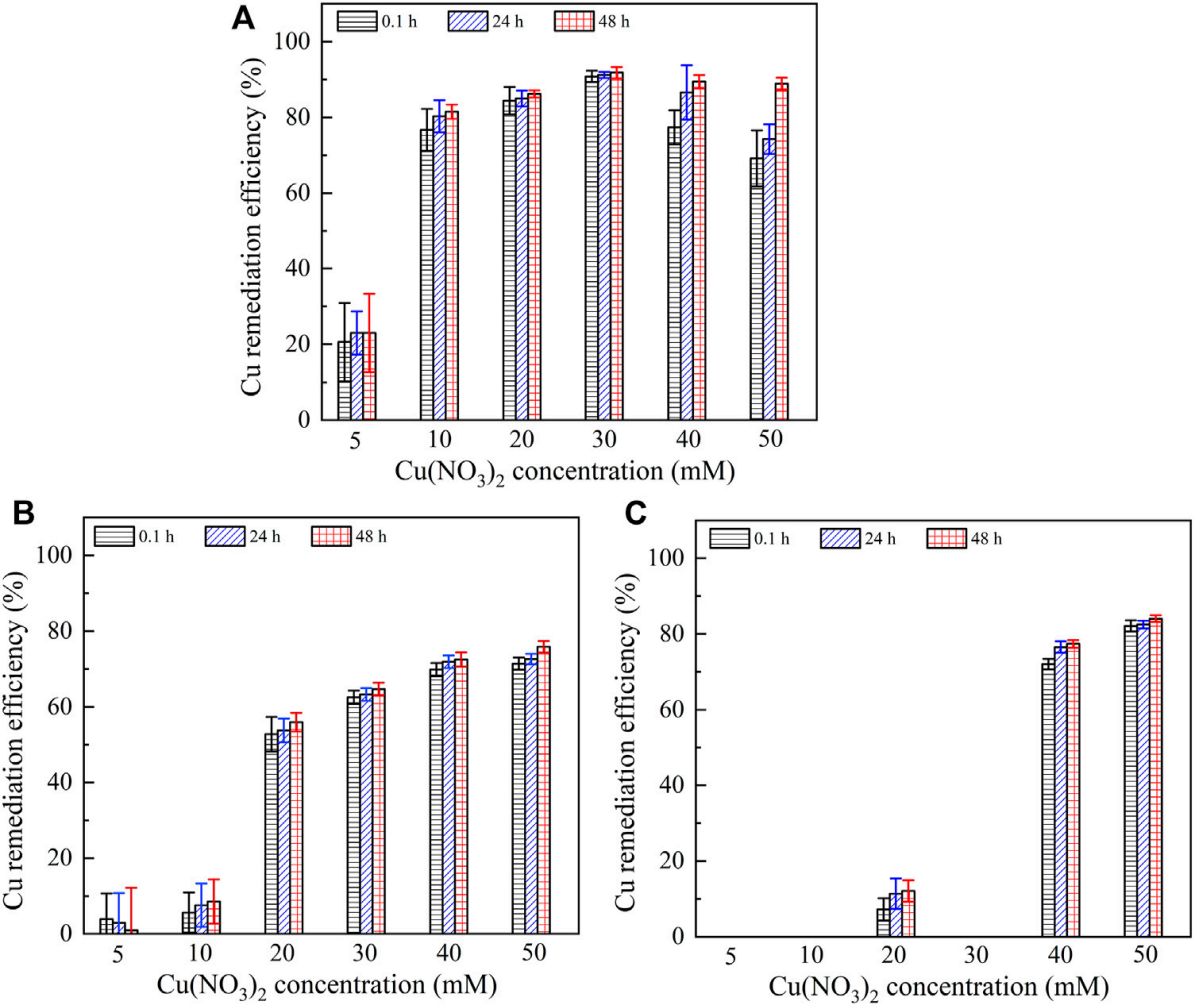
Relationship of remaining Cu^2+^ concentration and Cu remediation efficiency versus Cu(NO_3_)_2_ concentration: **(A)** without yeast extract and CaCl_2_, **(B)** with yeast extract, and **(C)** with yeast extract and CaCl_2_.

It is widely known that the lower the remaining heavy metal concentration after catalyzing urea hydrolysis and precipitating carbonate, the higher the remediation efficiency. Figure 7 presents the relationships of the remediation efficiency versus Pb(NO_3_)_2_ concentration after 0.1, 24, and 48 h. The remediation efficiency could be as low as 80% when subjected to no yeast extract and calcium source addition. It is increased to 92% under the effect of yeast extract and further to 100% under the effect of yeast extract and CaCl_2_ addition. It is worth noting that Pb^2+^ > 20 mM starts depressing the ureolytic bacteria, and therefore, there appears a sudden decline in the remediation efficiency when subjected to no yeast extract and calcium source addition. In contrast, a similar decline cannot be observed when subjected either to the effect of yeast extract or to the effect of yeast extract and CaCl_2_ addition. On the other hand, for a given concentration of Pb(NO_3_)_2_, the remediation efficiency is increased notably from 0.1 to 24 h, and its increase from 24 to 48 h becomes insignificant as before. These results are especially significant when subjected to no yeast extract and calcium source addition and are in good agreement with the measurements of NH_4_^+^ concentration, as shown in Figure 6. Figure 8 shows the relationships of the remediation efficiency versus Cu(NO_3_)_2_ concentration after 0.1, 24, and 48 h. The remediation efficiency of about 20% is measured when Cu(NO_3_)_2_ concentration = 5 mM, and it sharply increases to higher than 80% when Cu(NO_3_)_2_ concentration = 10 mM (see Figure 8A). The remediation efficiency continues to increase to as high as 92% when Cu(NO_3_)_2_ concentration = 30 mM, and then it decreases and remains constant when Cu(NO_3_)_2_ concentration falls in a 40–50 mM range. Under the effect of yeast extract, the remediation efficiency below 10% is measured when Cu(NO_3_)_2_ concentration falls in a 5–10 mM range, and it is increased up to approximately 80% when Cu(NO_3_)_2_ concentration = 50 mM (Figure 8B). The remediation efficiency under the effect of yeast extract and CaCl_2_ addition is measured to be below 10% when Cu(NO_3_)_2_ concentration falls in a 5–30 mM range (Figure 8C). It increases to about 77% when Cu(NO_3_)_2_ concentration = 40 mM and further to 84% when Cu(NO_3_)_2_ concentration = 50 mM. These results indicate that the remediation efficiency against Cu^2+^ is not as good as that against Pb^2+^, and its reduction appears to present in some specific ranges of Cu(NO_3_)_2_ concentration. Further exploration to identify the speciation and sequence of carbonate precipitation is considered of great necessity in order to reveal the mechanisms leading to the reduction in the remediation efficiency. In light of this, biomineralization simulations were conducted in the present work, and the simulated results are presented and analyzed as follows.

### Biomineralization Simulation

**FIGURE 9:**
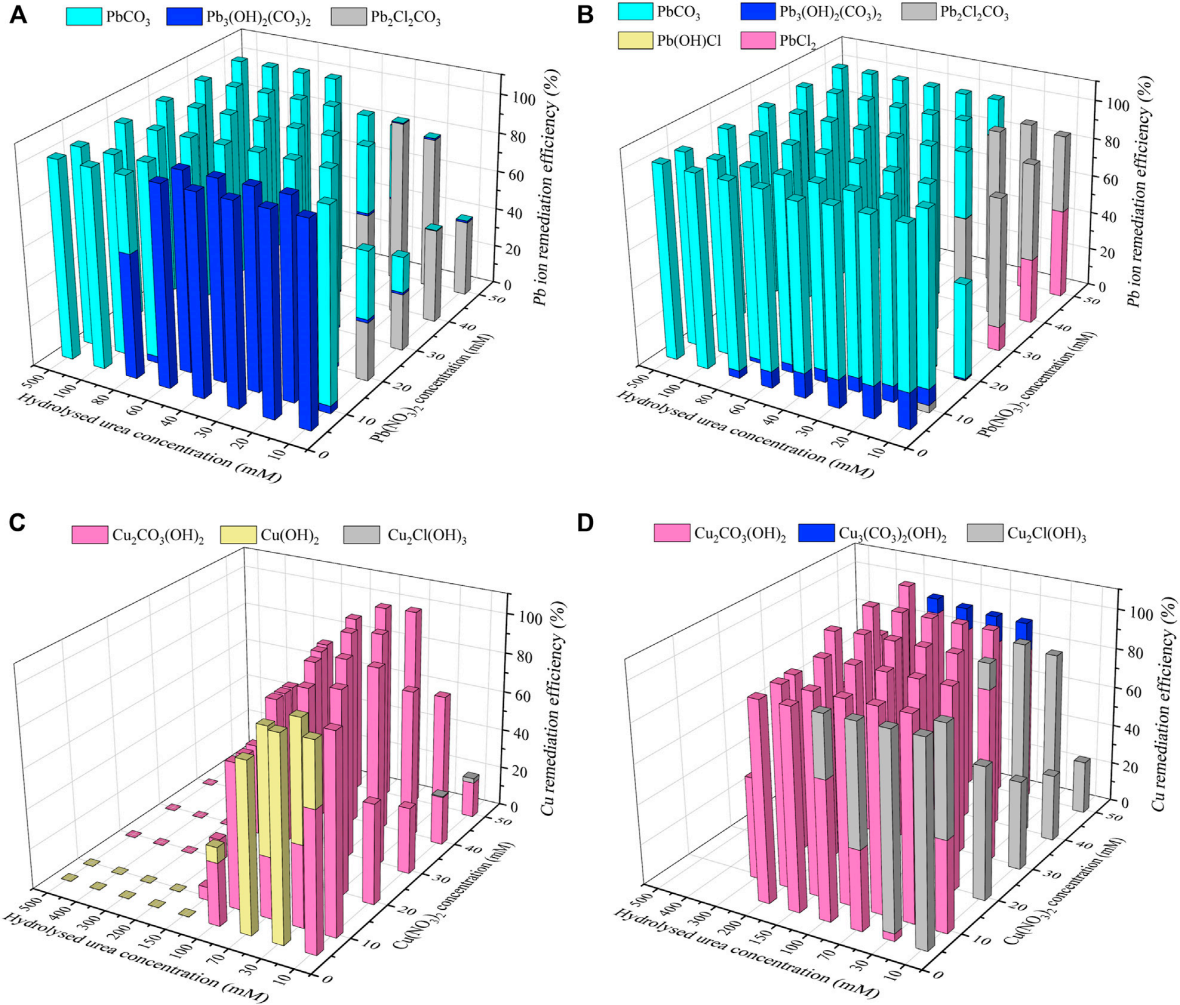
Variations of the Pb remediation efficiency against different Pb^2+^ concentration ranges considering hydrolysed urea concentrations varying in a 10–100 mM: **(A)** no calcium source addition; **(B)** with CaCl_2_ addition; variations of the Cu remediation efficiency against different Cu^2+^ concentration ranges considering hydrolysed urea concentrations varying in a 10–500 mM: **(C)** no calcium source addition; **(D)** with CaCl_2_ addition.

Carbonate precipitations present in the test tube experiments were simulated by Visual MINTEQ software toward investigating their speciation and sequence of precipitation. NH_4_^+^ and CO_3_^2-^ concentrations that are extracted upon the completion of urea hydrolysis in the test tube experiments are inputted in the biomineralization simulations. Furthermore, when the effect of yeast extract has already increased the degree of urea hydrolysis, the biomineralization simulations were simplified to those considering the effect of calcium source addition only. Figure 9A shows the relationships of the remediation efficiency versus the hydrolyzed urea concentration against Pb(NO_3_)_2_ concentrations varying within a 5–50 mM range, when subjected to no calcium source addition, in which the hydrolyzed urea concentration can be equivalent to the concentration of CO_3_^2−^ (i.e., degree of urea hydrolysis). When the hydrolyzed urea concentration is 500 mM, the degree of urea hydrolysis is 100% (Table 2). In contrast, the degree of urea hydrolysis corresponds to 0% when the hydrolyzed urea concentration is reduced to 0 mM. Given that the concentration of Pb(NO_3_)_2_ is 10 mM and the speciation of carbonate precipitation is recognized as Pb_3_(CO_3_)_2_(OH)_2_, 10 mM CO_3_^2−^ can precipitate 15 mM Pb^2+^ (= 10 × 3/2), which is in excess of 10 mM Pb^2+^ and also indicates the remediation efficiency being 100% (Figure 9A). When the discharge of CO_3_^2-^ does not reach a concentration that is required to precipitate Pb^2+^, the remediation efficiency is degraded where PbCl(OH), (PbCl)_2_CO_3_, and Pb_3_(CO_3_)_2_(OH)_2_ are precipitated (Figure 9A). In contrast, when subjected to the effect of CaCl_2_ addition, PbCl_2_, PbCl(OH), (PbCl)_2_CO_3_, and Pb_3_(CO_3_)_2_(OH)_2_ are precipitated when a lower concentration of the CO_3_^2−^ present causes difficulty in precipitating Pb^2+^ (Figure 9B). As the concentration of CO_3_^2-^ in the biomineralization process is high enough, the speciation of carbonate precipitation corresponds to PbCO_3_ under no calcium source addition and PbCO_3_ and CaCO_3_ under the effect of CaCl_2_ addition (see Eqs 6, 7). These results show that the speciation of carbonate precipitation varies not only with the concentration of CO_3_^2-^ but also with the calcium source addition. Figure 9C shows the relationships of the remediation efficiency versus the hydrolyzed urea concentration against Cu(NO_3_)_2_ concentrations varying in a 5–50 mM range, when subjected to no calcium source addition. They behave in an ascending manner in the first place and then in a descending manner, which differs from what we have seen in Figure 9A. Cu_2_Cl(OH)_3_ and Cu_3_(CO_3_)_2_(OH)_2_ are precipitated when low CO_3_^2−^ concentration precipitates a small number of Cu^2+^. In contrast, high CO_3_^2−^ precipitates the majority of Cu^2+^, and Cu_2_CO_3_(OH)_2_ is precipitated. Cu_2_Cl(OH)_3_, Cu_3_(CO_3_)_2_(OH)_2_, and Cu_2_CO_3_(OH)_2_, when under the effect of CaCl_2_ addition, are transformed with the increasing concentration of CO_3_^2−^ to CaCO_3_ (Figure 9D). The degradation in the remediation efficiency against Pb^2+^ presents under lower CO_3_^2−^ concentrations (corresponding to higher Pb^2+^ concentrations), whereas it vanishes or becomes minimal when subjected to higher CO_3_^2−^ concentrations (corresponding to lower Pb^2+^ concentrations) (Figures 9A,B). However, the degradation in the remediation efficiency against Cu^2+^ presents not only under lower CO_3_^2−^ concentrations but also under higher ones, indicating that it does not rely only on the concentrations of CO_3_^2−^ but on other influencing factors. They are worthy of investigation, revealing the mechanism affecting the remediation efficiency against Cu^2+^. As indicated by the measurements of pH, the higher the degree of urea hydrolysis, the closer the pH surrounding conditions to approximately 9, and the more significant the degradation in the remediation efficiency against Cu^2+^ (Figure 4B). In other words, the degradation in the remediation efficiency against Cu^2+^ is also attributed to pH surrounding conditions. However, the degradation in the remediation efficiency, under the effect of CaCl_2_ addition, appears not as significant as that under no calcium source addition. The effect of CaCl_2_ addition seems to ease the impact of pH surrounding conditions because in most cases, the remediation efficiency of approximately 100% is attained when the hydrolyzed urea concentration falls within a 0–300 mM range (corresponding to Cu^2+^ concentration range of 30–50 mM). This is due to the fact that Ca^2+^ which ionized from CaCl_2_ can react with CO_3_^2−^ to form CaCO_3_ precipitation, where CO_3_^2−^ is obtained by the reaction of CO_2_ produced by urea hydrolysis with OH^−^. A large amount of OH^−^ is consumed, reducing the pH value of the solution (see Eqs 1–6). The reducing pH causes a difficulty in promoting the formation of copper–ammonia complexes.

### Mechanisms Affecting Remediation Efficiency

Pb^2+^ or Cu^2+^ can notably affect the ureolytic bacteria and urease activity in the biomineralization process, leading to some difficulty in elevating the degree of urea hydrolysis and attaining a satisfactory remediation efficiency. Generally, the higher the concentration of Pb^2+^ or Cu^2+^, the lower the degree of urea hydrolysis, and lower the remediation efficiency. Furthermore, the effect of Cu^2+^ outweighing the effect of Pb^2+^ explains why the remediation efficiency against Cu^2+^ is much lower than that against Pb^2+^. On the other hand, the effect of yeast extract is proven effective in reducing the effect of Pb^2+^ or Cu^2+^ and causing the ureolytic bacteria to secrete urease for catalyzing urea hydrolysis. The experimental results indicate that the degradation in the remediation efficiency against Cu^2+^ is not only counted upon the degree of urea hydrolysis but also upon pH surrounding conditions. In the present work, a pH of approximately 9 leads to the lowest remediation efficiency. In light of this, attaining satisfactory remediation efficiency relies upon the degree of urea hydrolysis and pH surrounding conditions, while remedying Cu^2+^. When the degree of urea hydrolysis is high enough but the remediation efficiency is not as high as expected, a modification of pH surrounding conditions is considered to be of great necessity. Notwithstanding that, the simulated results indicate that the degradation in the remediation efficiency against Cu^2+^ presents when subjected not only to lower degrees of urea hydrolysis but also to higher degrees of urea hydrolysis. Such a high degree of urea hydrolysis turns pH surrounding conditions into highly alkaline environments, thereby modifying the speciation of carbonate precipitation (i.e. copper–ammonia complexes). The formation of copper–ammonia complexes turns Cu^2+^ into a free state toward degrading the remediation efficiency. The effect of CaCl_2_ addition eases the degradation in the remediation efficiency. It is worth noting that there appears a discrepancy in the remediation efficiency between the experimental and simulated results, most likely because of the neglection of dissolution of carbonate precipitation by the biomineralization simulations.

On the whole, attaining a satisfactory remediation efficiency relies generally upon the degree of urea hydrolysis. pH surrounding conditions, while remedying Cu^2+^, can significantly degrade the remediation efficiency. The effect of yeast extract prevents the ureolytic bacteria from losing their activity under heavy metal ion stress. The degradation in the remediation efficiency against Cu^2+^ also presents under higher degrees of urea hydrolysis. This is due to the fact that such a high degree of urea hydrolysis turns pH surrounding conditions into highly alkaline environments, thereby promoting the formation of copper–ammonia complexes and degrading the remediation efficiency. The effect of CaCl_2_ addition modifies pH surrounding conditions and further eases the degradation in the remediation efficiency. These results shed light on the importance of modifying pH surrounding conditions in capsulizing Cu^2+^ using the bioinspired calcium carbonate precipitation.

## Conclusion

This study investigated the effects of bacterial culture and calcium source addition on Pb and Cu remediation using the bioinspired calcium carbonate precipitation. Based on the results and discussion, some main conclusions can be drawn as follows:

1) The effect of Cu^2+^ outweighing the effect of Pb^2+^ explains why the remediation efficiency against Cu^2+^ is much lower than that against Pb^2+^. The lower the degree of urea hydrolysis, the lower the remediation efficiency. The effect of yeast extract reduces the effect of Pb^2+^ or Cu^2+^ on the ureolytic bacteria and urease activity.
2) Ca^2+^ forms competitive adsorption with Pb^2+^ and binds themselves with the ureolytic bacteria, enhancing the resistance against Pb^2+^. Ca^2+^ play different roles while remedying Cu^2+^, namely, modifying pH surrounding conditions, preventing the formation of copper–ammonia complexes, and securing the remediation efficiency.
3) The degree of urea hydrolysis might not be the most crucial factor in terms of the remediation efficiency against Cu^2+^. The findings shed light on the importance of modifying pH in capsulizing Cu^2+^ using the bioinspired calcium carbonate precipitation.

## Data Availability

The original contributions presented in the study are included in the article/Supplementary Material, further inquiries can be directed to the corresponding author.
